# First-Principle Prediction on STM Tip Manipulation of Ti Adatom on Two-Dimensional Monolayer YBr_3_

**DOI:** 10.1155/2019/5434935

**Published:** 2019-02-04

**Authors:** Pan Liu, Maokun Wu, Hui Liu, Feng Lu, Wei-Hua Wang, Kyeongjae Cho

**Affiliations:** ^1^Department of Electronic Science and Engineering and Tianjin Key Laboratory of Photo-Electronic Thin Film Device and Technology, Nankai University, Tianjin 300071, China; ^2^Department of Material Science and Engineering, The University of Texas at Dallas, Richardson 75080, USA

## Abstract

Scanning tunneling microscopy (STM) is an important tool in surface science on atomic scale characterization and manipulation. In this work, Ti adatom manipulation is theoretically simulated by using a tungsten tip (W-tip) in STM based on first-principle calculations. The results demonstrate the possibility of inserting Ti adatoms into the atomic pores of monolayer YBr_3_, which is thermodynamically stable at room temperature. In this process, the energy barriers of vertical and lateral movements of Ti are 0.38 eV and 0.64 eV, respectively, and the Ti atoms are stably placed within YBr_3_ by >1.2 eV binding energy. These theoretical predictions provide an insight that it is experimentally promising to manipulate Ti adatom and form artificially designed 2D magnetic materials.

## 1. Introduction

In 1959 at the American Physical Society meeting, Feynman has given a now famous lecture entitled “There's Plenty of Room at the Bottom,” and he has envisioned a possibility of atomic scale manipulation of materials [1]. Two decades later, scanning tunneling microscopy (STM) [2] was invented as the first step toward Feynman's vision, and STM has been a widely used experimental technique to characterize the surface structure and obtain high-resolution images on an atomic scale [3–7]. In 1989, researchers at IBM has used STM to arrange 35 xenon atoms on crystalline Ni surface to write “IBM” atomic logo, which has demonstrated the ultimate atomic scale manipulation of materials [8]. Subsequent to this demonstration, there are many atomic structure manipulations of surface such as Fe or Co atomic corrals on Cu (111) [9, 10]. Nowadays, STM is routinely used to manipulate atoms and molecules on the surface with specific configurations [11–15]. For example, it was predicted that the tungsten tip (W-tip) of STM equipment can induce the rearrangements of the atoms on the Si (100) surface [11, 12]. The manipulation of Cu adatom movement by Cu and Ag tips is induced on anisotropic Cu surfaces [13]. However, all the atomic manipulations are typically performed in ultrahigh vacuum (UHV) environment at a very low temperature (e.g., 4 K) due to the reactivity of the surface and high mobility of surface atoms. Owning to these limitations, the atomic scale manipulation still remains as a proof-of-concept demonstration of future technological possibility of building atomically precise structures along Feynman's vision. Practical applications of atomic manipulations require relatively inert surfaces (without requiring UHV) and stabilizing atoms on surface even at room temperature. Furthermore, it would be beneficial to utilize surfaces of two dimensional (2D) materials rather than the surface of bulk materials (e.g., previously used Ni, Cu, or Si surfaces) so that it would be possible to stack surface atomic structures along the vertical direction (i.e., 3D stacking). Even though these are desirable characteristics of atomically manipulated structures, there is no known materials or methods to achieve such goals.

In this work, we present here a new two dimensional (2D) atomic pore material as a promising substrate to develop stable atomic structures based on STM tip manipulations. Specifically, monolayer YBr_3_ with a large band gap of 4.20 eV, which was reported in our previous modeling work [16], is selected to provide stable atomic sites. Similar to MoS_2_, YBr_3_ is a layer material formed by 2D materials with van der Waals stacking with the dimension reduction from three-dimensional (3D) to 2D for single layer YBr_3_. In comparison with 2D transition metal dichalcogenide (TMDs) materials, MX_2_ represented by MoS_2_, YBr_3_ can be regarded as Y_2/3_□_1/3_Br_2_, where □ denotes empty sites, leading to a unique 2D atomic porous structure. Monolayer YBr_3_ has a pore diameter of 5.29 Å, which provide stable sites to introduce foreign atoms into the cavities. Therefore, YBr_3_ (more generally MX_3_ class-layered materials) provide a promising platform of inert surface with high-density pore sites which may stably accommodate adatoms manipulated by STM tip.

Pristine monolayer YBr_3_ is nonmagnetic since no localized magnetic moments are formed at Y or Br sites. If a transition metal (TM) atom could be stably introduced into the pore, then an artificial 2D magnetic material may be manufactured similar to Co atomic corral on Cu (111) surface, but with stability in ambient condition at room temperature rather than in UHV at 4 K. Thus, it is significant to propose a possible scheme to insert a TM atom into the pore and to examine the theoretical feasibility using accurate density functional theory calculations. With this motivation, our present work is aiming to design an artificial 2D magnetic material based on monolayer YBr_3_ by STM tip manipulation. Here, we theoretically utilized a W-tip to manipulate titanium (Ti) atom on the surface of monolayer YBr_3_ using the first-principle calculation method. The properties of Ti-doped monolayer YBr_3_ are firstly explored. It is found that the Ti atom could stably locate in the pore and produce a localized magnetic moment. Then, the manipulations of Ti atom on monolayer YBr_3_ using W-tip (4-atom and 10-atom W-tip) were investigated in detail. The results show that the W-tip successfully leads to the Ti atom into the atomic pore of monolayer YBr_3_ and do not affect the electronic properties of Ti/YBr_3_ system.

## 2. Computational Details

**Figure 1:**
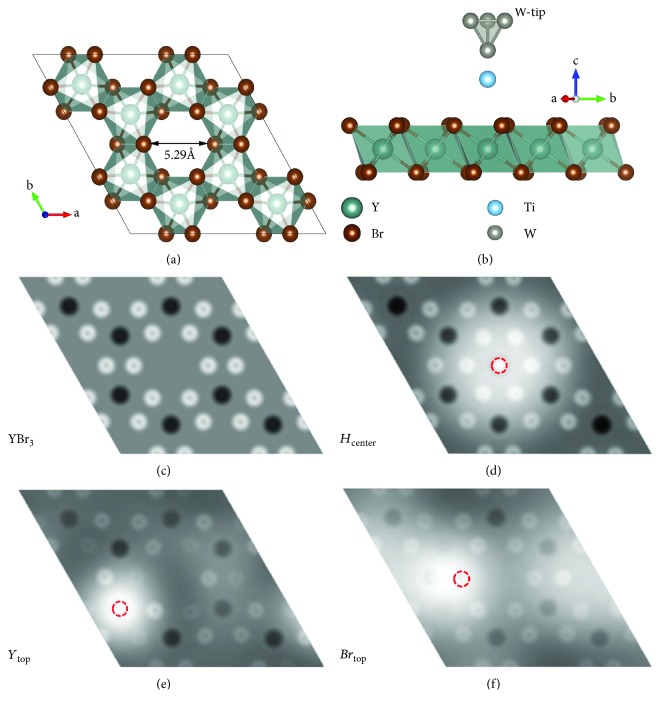
(a) The top view of crystal structures of monolayer YBr_3_. The double arrow indicates the pore size (5.29 Å) of monolayer YBr_3_. (b) The side view of W-tip manipulates the Ti atom on monolayer YBr_3_ surface. Theoretical simulation of STM images for (c) pristine YBr_3_, Ti atom on (d) *H*_center_, (e) *Y*_top_, and (f) *Br*_top_ site. Bright places denote the position of the Br and Ti atoms, and Y atoms are shown in the dark region. The Ti atomic positions are also outlined by red dashed circles.

The first-principle calculations based on density functional theory (DFT) method within Vienna ab initio simulation package (VASP) [17] were performed in all calculations. The generalized gradient approximation (GGA) with Perdew-Burke-Ernzerhof (PBE) [18] functional was adopted for the exchange-correlation potential. The plane-wave cutoff energy was set as 500 eV. The vacuum layer was set as a thickness of 30 Å along the *z* direction in order to minimize the interaction between the periodic layers. A 2 × 2 supercell in *ab* plane was used and the *k*-point mesh in the Brillouin zone was chosen as 5 × 5 × 1. The tip composed of four W atoms with tetrahedral structure was used to simulate the apex of STM tip. The W-tip was placed in the vacuum region with an apex atom pointing towards the surface of monolayer YBr_3_. Three tungsten atoms at the top of the tip were fixed (to represent the rest of STM tip) in one plane parallel to monolayer YBr_3_ plane as shown in Figure 1(b). To keep a different fixed tip-surface distance, the *z* positions of two Y atoms away from the pore and the top W atoms are fixed in each calculation step. The Ti atom was first placed directly below the bottom W atom at a distance of ~2 Å, and their *z* positions were relaxed. The climbing image nudged elastic band (CI-NEB) method [19, 20] was employed to simulate the manipulations of the Ti atom on monolayer YBr_3_ by using W-tip. In CI-NEB calculations, the energy convergence is 10^−4^ eV, and the force on each atom is less than 0.05 eV/Å. The energy convergence is 10^−5^ eV in the electronic structure calculations.

## 3. Results and Discussion

### 3.1. The Geometrical Structure of Monolayer YBr_3_ and Theoretical Simulation of STM Images

As shown in Figure 1(a), the atomic porous structure is induced due to the unoccupied 1/3 Y site in monolayer YBr_3_ compared to MX_2_-type 2D materials. Monolayer YBr_3_ has a relatively large pore diameter of 5.29 Å, which implies the possibility of introduction of foreign atoms into the pores. In this work, the Ti atom is taken as an example for adatom manipulation by STM tip. First, the favorable Ti adatom configurations should be clarified in the absence of W-tip. Four high-symmetric adatom binding sites are considered in Figures 1(d)–1(f), i.e., hollow site in the center of the pore (*H*_center_ site), top site directly above a Y atom (*Y*_top_ site), top site directly above a Br atom (*Br*_top_ site), and another hollow site above the pore (*H*_top_ site, not shown here). To identify the most stable configuration, the total energies of four doping sites for Ti/YBr_3_ system are calculated. The total energies of Ti/YBr_3_ system with *Y*_top_ and *Br*_top_ sites have higher energies than that of *H*_center_ site by 1.20 and 2.20 eV, respectively. It is noted that when a Ti atom is directly placed above the pore (*H*_top_ site), the Ti at this site is unstable and would move into the center of the pore (*H*_center_ site). Therefore, the most stable configuration is *H*_center_ site. The formation energy (*E_f_*) of Ti/YBr_3_ system was defined as *E*_*f*_ = *E*(Ti/YBr_3_) - *E*(YBr_3_) - *E*(Ti), where *E*(Ti/YBr_3_), *E*(YBr_3_), and *E*(Ti) are the energies of Ti/YBr_3_ system, pristine monolayer YBr_3_, and Ti atom, respectively. The obtained formation energy of Ti/YBr_3_ system is -4.80 eV, indicating the stable state of the doping system relative to the isolated Ti atom and YBr_3_ monolayer. The introduced Ti atom into the pore of monolayer YBr_3_ produces a localized magnetic moment of ~1.97 *μ*_B_, making this system to be a promising 2D magnetic material.

Even though the stability of Ti-doped YBr_3_ as an artificial 2D magnetic material has been examined above, the effective scheme to realize the controlled TM doping in YBr_3_ is yet to be proposed for the guidance of further experiment validation. The atomic manipulation using STM technique is a powerful way to achieve controlled atomic arrangements onto the surface. Here, the constant height mode STM images of Ti-doped monolayer YBr_3_ in Figures 1(c)–1(f) are theoretically simulated and acquired, which provide the references for further experimental characterization of the atomic structures of Ti/YBr_3_ system. The brightest region in Figures 1(d)–1(f) denotes the location of the Ti atom, where the atomic positions can be identified.

### 3.2. The Manipulation of Ti Atom on Monolayer YBr_3_ Using W-Tip

**Figure 2:**
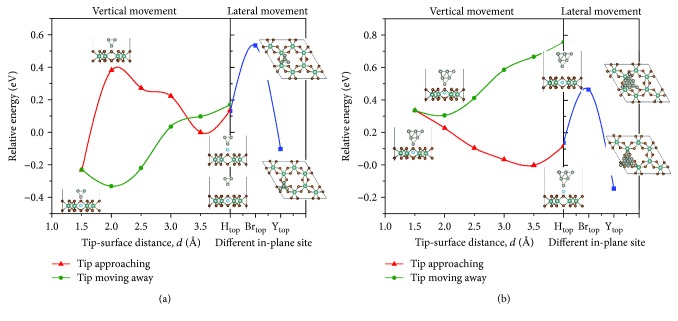
The relative energies of (a) 4-atom W-tip and (b) 10-atom W-tip controlled Ti atom on monolayer YBr_3_ in the lateral (right) and vertical (left) movements for W-tip approaching and moving away process. Here, the lowest total energy in W-tip approaching process for *d* = 3.5 Å is set as the reference energy. The inserted images denote the geometric structures under different tip-surface distances.

Next, we examine the Ti adatom manipulation on YBr_3_ surface to identify a pathway to place the Ti atom at the stable *H*_center_ site. The Ti atom doping process into YBr_3_ using W-tip could be schematically described by throwing away the Ti atom by W-tip/Ti system to the YBr_3_ surface due to the competition between the Ti interactions with W-tip and YBr_3_. The distance between the apex of W-tip and top Br-plane of YBr_3_ was defined as the tip-surface distance, *d*. The initial tip-surface distance was set as 4 Å. The total energies of W-tip/Ti system on *Y*_top_, *Br*_top_, and *H*_top_ site were calculated (Figure 2(a) right). Compared with *Br*_top_ and *H*_top_ sites, W-tip/Ti on *Y*_top_ site is relatively more stable indicating significant interactions among W-tip, Ti adatom, and surface at this distance. The presence of W-tip has reduced the relative stability of Ti at *Y*_top_ vs. *Br*_top_ from 1.0 eV to 0.64 eV leading to the possibility of W-tip manipulation of later motion of Ti. Since *H*_center_ site is most stable for an individual Ti atom on YBr_3_, the lateral movement of W-tip/Ti along *Y*_top_-*Br*_top_-the top site of the pore is the first step to manipulate Ti atom between different sites on YBr_3_.

When the W-tip picks up a Ti adatom on the surface and the W-tip/Ti moves above the pore of YBr_3_, the next process is to place the Ti atom in the pore at *H*_center_ site. The placement process could be divided into two tip vertical movements. The first step is the W-tip/Ti approaching to the surface and push the Ti atom toward the center of the pore. The second step is the W-tip moving away and leaving the Ti atom within the pore, which completes the Ti adatom placement at the stable *H*_center_ site. In the vertical movement processes, the total energy was calculated at each step and the relative energies were illustrated in Figure 2(a) left. In the process of W-tip approaching the surface, the W-tip moves together with the Ti atom until the tip-surface distance is ~2.0 Å (Ti atom at the top Br-plane) with increasing energy indicating that energy is required to push Ti atom into the pore of YBr_3_. However, when the tip-surface distance is further decreased to ~1.5 Å, the Ti atom falls into the pore and the total energy is much reduced owing to the strong binding of Ti with its surrounding Br and Y atoms. Note that the energy barrier is ~0.38 eV which corresponds to the transition probability of exp(-0.38 eV/k_B_T) = 4.2 × 10^−7^ at room temperature (T = 300 K). Since the final state is thermodynamically more stable, the time scale of thermally activated transition would be *τ* = 10^−12^/(4.2 × 10^−7^) = 2.4 × 10^−6^ s = 2.4 *μ*s (using the attempt frequency *ν* = 10^12^ s^−1^). Consequently, as the STM tip moves down in *μ*s scale, thermal fluctuations would bring the Ti adatom to the stable *H*_center_ site even without the externally provided force to overcome the barrier. Now, the final step is to remove the W-tip without pulling the Ti atom along with it. Note that the total energy curve of the W-tip moving away from the surface is different from the tip approaching one due to the different locations of Ti atom which is stably bonded at *H*_center_ site. With the W-tip moving away, the interaction between W-tip and Ti atom decreases and the total energy gradually enhances without additional activation barrier. Through the whole process, the W-tip manipulates the Ti atom and finally inserts it into the pore of monolayer YBr_3_. As noted earlier, Ti at *H*_center_ site is more stable than *Y*_top_ and *Br*_top_ sites by 1.20 and 2.20 eV, these energy differences indicate that the Ti atoms can maintain the position for the minimum time scale of *τ* = 10^−12^ × exp(1.2 eV/k_B_T) = 1.4 × 10^8^ s (4.6 years) at room temperature.

**Figure 3:**
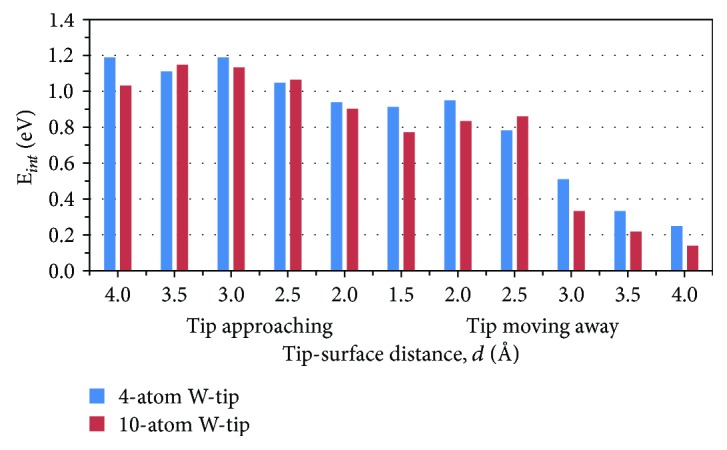
The tip-surface interaction energies for 4-atom W-tip and 10-atom W-tip in the vertical movement. All the interaction energies are negative; here, the absolute values are plotted.

As mentioned above, W-tip firstly came with a Ti atom (W-tip/Ti), then put the Ti atom into the atomic pore, and then the tip moved away from the monolayer leaving the Ti atom to stay in the pore (Ti/YBr_3_). Therefore, the tip-surface interaction energy could be defined as *E*_int_ = *E*(W-tip/Ti/YBr_3_) - *E*(W-tip/Ti) - *E*(YBr_3_) for tip approaching process and *E*_int_ = *E*(W-tip/Ti/YBr_3_) - *E*(W-tip) - *E*(Ti/YBr_3_) for tip moving away process. *E*(W-tip/Ti/YBr_3_) is the total energy of W-tip/Ti/YBr_3_ system. *E*(W-tip/Ti) and *E*(YBr_3_) were the separately calculated energies for W-tip/Ti system and YBr_3_ in the tip approaching process. *E*(W-tip) and *E*(Ti/YBr_3_) were the separately calculated energies for W-tip and Ti/YBr_3_ system in the tip moving away process. The tip-surface interaction energies for 4-atom W-tip in the vertical movement were plotted in Figure 3. When W-tip coming with Ti atom, W-tip/Ti system had larger interaction with monolayer YBr_3_ than that of between W-tip and Ti/YBr_3_ system in the tip moving away process. It should be noticed that the two energies of tip approaching and moving away have very small difference at the tip-surface distance around 3 Å in Figure 2(a). While, the interaction energies is -1.19 and -0.51 eV, respectively. Ti atom bonded with W-tip and interacted with monolayer YBr_3_ in the approaching process. In the opposite process at the distance of ~3 Å, the moving away of W-tip had a relatively weak influence on Ti/YBr_3_ system. With the increase of tip-surface distance, the interaction between W-tip and Ti/YBr_3_ system decreased, indicating the successful manipulation of Ti atom into the atomic pore of monolayer YBr_3_.

In order to examine the effect of a larger W-tip on Ti atom manipulation, a W-tip with 10 atoms in pyramidal model was used here [21]. The relative energies of 10-atom W-tip controlled Ti atom on monolayer YBr_3_ in the lateral and vertical movements for W-tip approaching and moving away process were illustrated in Figure 2(b). In the lateral movement, the relative energy of Ti at *Y*_top_ vs. *Br*_top_ is 0.61 eV, a slightly difference from the 4-atom W-tip results. In the tip approaching movement, the energy reduce first and then increase with the tip-surface distance reducing from 4.0 to 1.5 Å. The energy reaches the highest value when Ti was in the pore and the barrier is 0.34 eV, different from the lowest energy in 4-atom W-tip. Although Ti atom was put into the atomic pore, it interacted with W-tip and did not stay in Y-plane like 4-atom W-tip results, which induced the high energy. With the W-tip moving away, the relative energy first reduce in 2 Å and then increase with the tip-surface distance increase, the same behavior as that of 4-atom W-tip. The energy of whole system increased and tip-surface interaction energies decreased with the increase of tip-surface distance (shown in Figure 3), evidencing the possibility to manipulate Ti atom into the atomic pore of monolayer YBr_3_ by using 10-atom W-tip.

**Figure 4:**
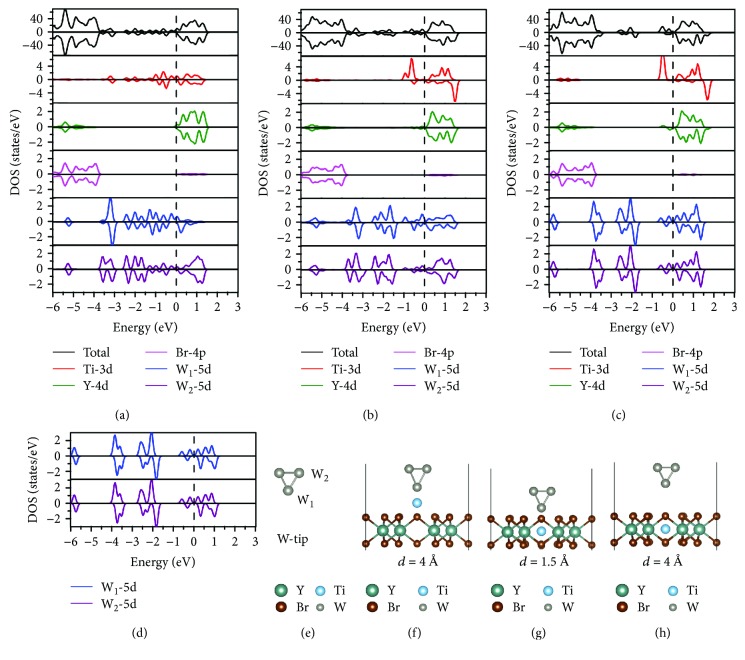
Total and partial density of states (PDOS) and geometric structures of W-tip/Ti/YBr_3_ system in the W-tip approaching process for (a), (f) *d* = 4 Å and (b), (g) *d* = 1.5 Å. (c), (h) PDOS and geometric structure of W-tip/Ti/YBr_3_ system in the W-tip moving away process for *d* = 4 Å. (d), (e) PDOS and geometric structure of pristine W-tip. The Fermi level indicated by the black dashed lines is set to zero.

Finally, we will examine the electronic structure changes during the W-tip manipulation of Ti adatom on YBr_3_ surface. Here, 4-atom W-tip manipulated results were taken as an example. In order to examine the effect of the W-tip on the electronic structures of Ti-doped YBr_3_ system, the total and partial density of states (PDOS) of pristine W-tip and W-tip/Ti/YBr_3_ were displayed in Figure 4. In the process of tip approaching the surface at *d* = 4 Å, Figure 4(a) shows the interaction between Ti atom and W-tip. To analyze the electronic structure data, we first calculate the electronic structure of an isolated W-tip (Figure 4(d)). As shown in Figure 4(e), the W atoms in W-tip could be classified into two classes, the apex atom (W_1_) and the top atoms (W_2_), and their PDOS are identical for an isolated W-tip due to the symmetry. When the tip-surface distance is at 1.5 Å (Figure 4(g)), the PDOS in Figure 4(b) of the Ti atom shows an evident different profile and spin polarization relative to that in Figure 4(a). This difference indicates that the interaction between Ti and its surrounding Br and Y atoms occurs in this case. It is also found that the PDOS of the W-tip in W-tip/Ti/YBr_3_ system in Figure 4(c) (corresponding to Figure 4(h) geometry) is very similar to that of pristine W-tip in Figure 4(d), suggesting that the influence of the W-tip on Ti-doped YBr_3_ system could be neglected at the W-tip is shifted to YBr_3_-surface ~4 Å. Thus, the placement of Ti atom into the pore of YBr_3_ with the help of the W-tip utilizes the balance of bonding strengths of W-tip/Ti and Ti/YBr_3_ during the lowering and subsequent removal of the W-tip. It can also be seen from Figure 4(b) with Ti stayed in the atomic pore, there was spin splitting near the Fermi level, which indicates the Ti-induced magnetic moment of ~1.97 *μ*_B_.

## 4. Conclusions

In summary, we have theoretically simulated the Ti adatom manipulation on the monolayer YBr_3_ surface by using the W-tip of STM. The stable configuration of Ti is right in the pore of YBr_3_, i.e., *H*_center_ site. Combing the results of the relative energies and the electronic structures in the whole process, it is shown that this Ti placement strategy in monolayer YBr_3_ using W-tip is feasible. Moreover, the doped Ti possesses a localized magnetic moment (~1.97 *μ*_B_), which forms a promising basis to design artificial 2D magnetic materials using metal halides MX_3_.

## Data Availability

The data used to support the findings of this study are available from the corresponding author upon request.
